# Jejunojejunal Intussusception in an 11 year old: A Case Report

**DOI:** 10.31729/jnma.9196

**Published:** 2025-08-31

**Authors:** Samir Karki, Jasmine Bajracharya, Ashish Pyakurel, Ganesh Jaishi, Ashima Kafle

**Affiliations:** 1 Prime Health Care Center, Sukedhara, Kathmandu, Nepal; 2 Department of General Surgery, Nepal Medical College, Kathmandu, Nepal; 3 Nepal Medical College, Kathmandu, Nepal

**Keywords:** *abdominal pain*, *conservative*, *intussusception*, *jejunal diseases*

## Abstract

Intussusception is a condition which usually occurs below the age of five. Here we present a case report of an 11 year old boy who presented with abdominal pain. Diagnosis was confirmed by Computed tomography imaging which showed a Jejunojejunal intussusception. Conservative management was done which led to a successful outcome. This case focuses on the need for considering intussusception in differential diagnosis in patients of age group above 6 years in diagnosis of abdominal pain and obstruction.

## INTRODUCTION

Intussusception is a condition where inward folding of one part of the intestine into another occurs with global incidence varying widely. About 74 intussusceptions occur per 100,000 infants, with higher rates observed in countries such as Japan (185) and Vietnam (302) whereas lower in India (18) and Bangladesh (9).^1^

It is a leading cause of intestinal obstruction below age 5 years, with ileocolic type in 80% and jejunojejunal type in 2.5% cases.^2-4^ Most intussusceptions occur in infants under 1 year and most small bowel intussusceptions are diagnosed late and often require surgery.^1,6^

This highlights the need to consider intussusception in older children and potential for non-surgical management in stable cases.

## CASE REPORT

We report a case of an 11 year old boy who presented to the Nepal Medical College Teaching Hospital Outpatient Department with a chief complaint of abdominal pain for 7 years. The pain , which was around the umbilical region, was acute in onset, dull aching, non radiating and had no aggravating and relieving factors.

The patient had a history of Abdominal tuberculosis 7 years back for which he was admitted in a hospital for 16 days. He was under Anti tubercular therapy for 6 months. Since then he experienced occasional abdominal pains every few months for which he would visit the Pediatrics OPD. He would receive symptomatic management with antispasmodics like Hyoscine butylbromide and Drotaverin, acid suppressants such as Ranitidine and Digestive enzyme preparations. Despite this, the pain recurred intermittently.

On examination, his vitals were stable. Palpation of his abdomen showed a lump of 2×2 cm^2^ in size over the right side of his umbilicus. The swelling was smooth , movable but showed no movement with respiration. On percussion, a tympanic note was heard and bowel sounds were present on auscultation. Hemoglobin level, Leucocyte count, Platelet count, Serum Urea, Serum creatinine, Sodium and Potassium levels were within normal limits.

A computed tomography abdomen was done which revealed a telescoping of about 2.8cm of a bowel segment involving a jejunal loop into another jejunal loop in the left upper abdominal cavity. The telescoping was located at the level of L3 vertebra, at about 33.2cm distal to the duodenojejunal flexure. Other findings that were noted include - a Bifid renal pelvis. Left extrarenal pelvis was present. A scoliosis was also noted which had a convexity towards left side. Cobb’s angle was 12 degrees.

The patient was admitted to the hospital for 6 days where he showed no signs of bowel obstruction. He was given He was managed conservatively with Dextrose and Sodium Chloride infusion , Injection Ornidazole, Injection Amikacin, Injection Pantoprazole and Injection Ondansetron. For the first 2 days, he was kept Nil Per Oral (NPO). On the 3rd day, he was started on a liquid diet, which advanced to soft diet the following day.

A pediatric consultation was done, which advised the continuation of the same treatment. No active intervention was carried out. However, Syrup Lactulose was added, which was to be taken twice daily once oral feeding was established. It was added as a therapeutic and prophylactic measure to soften the stool, ensure smooth bowel movements prevent any aggravation of the intussusception during symptomatic management.

**Figure 1:**
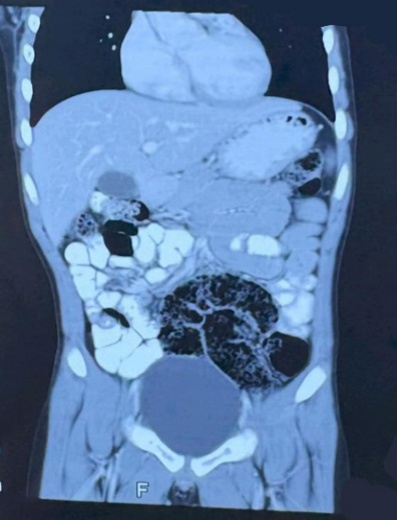
Computed Tomography Scan of the Abdomen of coronal view showing sausage shaped bowel loops, consistent with intussusception.

**Figure 2:**
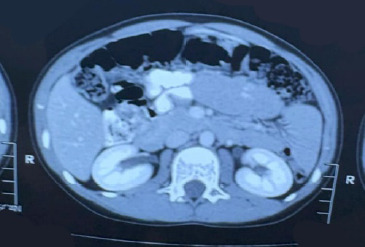
Computed tomography scan of the abdomen showing dilated bowel loops with mucosal folds.

**Figure 3:**
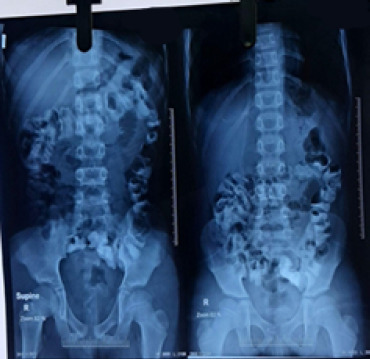
Abdominal plain X-ray showing dilated bowel loops, indicating bowel obstruction

**Figure 4:**
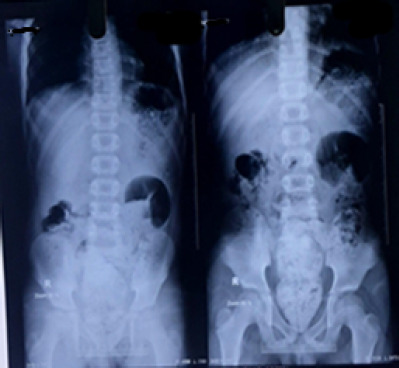
Abdominal Xray with contrast showing features of small bowel obstruction.

He was discharged with stable vitals. At the time of discharge, we advised him to take a high fiber diet and plenty of fluids. He was discharged with the following medications, each to be taken for 5 days-Tab Cefixime, Syrup Metronidazole Benzoate, Syrup Granisetron , Syrup Lactulose.

At the follow up which was done after 2 months, the patient showed no recurrence of symptoms. The patient returned to full normal activities. He had normal bowel, bladder habits and his diet was normal as well. Physical examination showed no abnormalities.

## DISCUSSION

Data focused on jejunojejunal intussuception in Nepal are scarce. A study done by Rajkarnikar et al. demonstrated that 267 (14.96%) of the admitted patients had intussuception.^7^ A study from the department of Pediatric surgery at B.P. Koirala Institute of Health Sciences, reported that the peak incidence of intussception occurred between the age of 4 and 7 months. Most cases occurred in less than 1 years of age.^8^

Our patient also had a bifid renal pelvis with an extrarenal component and a scoliosis with a left sided convexity (Cobb’s angle 12 degree). Literature suggests that such anomaly could occur as a part of syndromes such as Klippel-Feil and VACTERL , however there is no evidence to suggest that they could contribute to the lead points causing intussusception.

Intussusception manifests across various groups in different ways. Children usually present with characteristics features of abdominal pain , bloody stool and a mass that can be palpated on abdominal examination.^9,10^ In our case, the presentation was atypical as the abdominal pain had a duration of about 7 years and had no signs of bowel obstruction. Moreover, the presentation can be acute, intermittent or chronic.^11^ In this case , the patient symptom was chronic as he had abdominal pain for 7 years. Around 90% of pediatric intussusception cases are without a clear cause.^12^ In our case a definitive cause of the intussusception was not discovered despite a Computed tomography scan of the abdomen. This can be seen as a limitation in our approach.

With a computed tomography scan, the nature of the mass and its position can be defined with its relationship to adjacent structures and also can assist in staging when malignancy is suspected as cause of the intussusceptions.^10^

Kim et al recently reported that computed tomography of abdomen was able to distinguish between intussusception with and without a lead point. Intussusception with lead point demonstrated obstruction in bowel , edema of wall of the bowel, impairment in mesenteric circulation and presence of lead mass. In contrast, intussusception without a lead point showed no signs of obstruction and the target like or sausage-shaped mass with layering effect.^11^ These findings are consistent with the findings in our case, where there was no visible lead point.

In a study of 35 cases of isolated small bowel intussusception in children, patients with self limiting intussusception had a mean intussusception length of1.9cm, whereas those requiring surgical intervention had a mean length of 7.3cm. An intussusception length greater than 3.5 cm was identified as a strong independent predictor of the need for surgical intervention.^13^ If there is short segment intussusception with no identifiable lead point , careful observation is recommended instead of immediate intervention.^14^ Although it was previously thought that surgical intervention is universally appropriate, recent literature shows that for idiopathic intussusception, with low probably of malignancy can be managed conservatively.^15^ In contrast, other case reports have employed different management strategies. For instance, a 14-year-old girl with jejunojejunal intussusception caused by a jejunal polyp underwent laparotomy, bowel resection, and anastomosis.^2^

In our case, it was revealed a telescoping of about 2.8cm of a bowel segment involving a jejunal loop into another jejunal loop in the left upper abdominal cavity. He was managed conservatively . Broad spectrum coverage with Ornidazole and Amikacin in accordance with the hospital protocol was done as a precautionary measure against infections which could occur due to bowel ischemia or necrosis due to the intussusception. However, a systematic review suggests that the prophylactic use of antibiotics may not decrease the risk of complications and is unnecessary in hemodynamically stable children.^16^

Not all of the patients require therapeutic reduction. Small bowel intussusception can resolve on its own sometimes and air enema might fail to reach the particular site. The clinical presentation of patients should guide the treatment approach. ^17^ This approach highlights the strength in our approach to this case.

In the follow up, there was no recurrence. In the pediatric population, small bowel intussusception that resolves on its own is not uncommon making up as much as 17% of all cases of intussusceptions.^17,18^

This case emphasizes on the importance of including intussusception in the differential diagnosis of pediatric bowel obstruction despite its rarity. It also emphasizes on the possibility of a spontaneous resolution of intussusception. The best treatment options should be based on the patient’s symptoms. This case report emphasizes the need for patient centered strategy to ensure optimal outcome and minimize unnecessary interventions.

## Consents:

JNMA Case Report Consent Form was signed by the patient and is attached with the patient’s chart.
